# Michigan State Board of Examiners

**Published:** 1892-03

**Authors:** 


### MICHIGAN STATE BOARD OF EXAMINERS.

"No diplomas are recognized from any institution where requirements for graduation are not up to that of the University of Michigan."

This is the law of Michigan, according to a report on another page.

It would be well for colleges to commence at once the study of this department of Michigan University, or else decline to accept students from that State. When will the era of absurd laws cease ?

